# Remdesivir for Early COVID-19 Treatment of High-Risk Individuals Prior to or at Early Disease Onset—Lessons Learned

**DOI:** 10.3390/v13060963

**Published:** 2021-05-22

**Authors:** Lars Dölken, August Stich, Christoph D. Spinner

**Affiliations:** 1 Institute for Virology and Immunobiology, Julius-Maximilians-University Würzburg, 97078 Würzburg, Germany; 2 Helmholtz-Institute for RNA-Based Infection Research, 97080 Würzburg, Germany; 3 Department of Tropical Medicine, Klinikum Würzburg Mitte, 97074 Würzburg, Germany; august.stich@medmissio.de; 4 Technical University of Munich, School of Medicine, University Hospital Rechts der Isar, Department of Internal Medicine II, 81675 Munich, Germany; 5 German Center for Infection Research (DZIF), Partner Site Munich, Germany

**Keywords:** COVID-19, SARS-CoV-2, antiviral treatment, remdesivir

## Abstract

After more than one year of the COVID-19 pandemic, antiviral treatment options against SARS-CoV-2 are still severely limited. High hopes that had initially been placed on antiviral drugs like remdesivir have so far not been fulfilled. While individual case reports provide striking evidence for the clinical efficacy of remdesivir in the right clinical settings, major trials failed to demonstrate this. Here, we highlight and discuss the key findings of these studies and underlying reasons for their failure. We elaborate on how such shortcomings should be prevented in future clinical trials and pandemics. We suggest in conclusion that any novel antiviral agent that enters human trials should first be tested in a post-exposure setting to provide rapid and solid evidence for its clinical efficacy before initiating further time-consuming and costly clinical trials for more advanced disease. In the COVID-19 pandemic this might have established remdesivir early on as an efficient antiviral agent at a more suitable disease stage which would have saved many lives, in particular in large outbreaks within residential care homes.

## 1. Introduction

With the rise of the SARS-CoV-2 pandemic, substantial hope was initially placed in repurposing existing antivirals, in particular the nucleoside analog remdesivir, for the potential treatment of COVID-19 [1]. This RNA polymerase inhibitor was one of the most promising drugs that was initially developed to combat Ebola [2]. Remdesivir is a prodrug that is metabolized in the cell to the active triphosphate. This is then utilized for RNA synthesis instead of ATP by the viral RNA-dependent RNA polymerase. However, incorporation does not lead to strand termination, as is the case with many other nucleoside analogues. Rather, three more nucleotide building blocks are incorporated into the growing RNA chain before RNA synthesis comes to a halt [3]. The inhibitory effect of remdesivir can thus be partially reversed and overcome by the viral proofreading exoribonuclease nsp14 (nonstructural protein 14) [4], which renders remdesivir less efficient [5]. Nevertheless, remdesivir demonstrated potent inhibition of SARS-CoV-2 replication in vitro [3]. A number of clinical studies and its extensive clinical usage demonstrated that it can interfere with productive virus infection and mitigate the disease [6,7,8]. However, in stark contrast to the excellent antiviral activity of remdesivir in cell culture models [9], its effectiveness on the overall mortality of critically ill patients has not been shown. Accordingly, the World Health Organization (WHO) no longer recommends the use of remdesivir for the treatment of COVID-19 [10], while other guidelines restrict its use to the early stage of COVID-19 [11].

## 2. Result and Discussion

The clinical benefits observed with remdesivir show striking similarities to the known effects of the neuraminidase inhibitor oseltamivir, which is used for treating influenza A virus (IAV) infections. Despite oseltamivir’s high antiviral activity against IAV in vitro, large clinical studies have demonstrated that the drug has no measurable benefit on the course of disease if treatment is initiated later than two days after the onset of symptoms [12]. Starting treatment on the day of symptom onset or one day thereafter shortens the duration of symptoms by approximately two days and one day, respectively. Thus, oseltamivir is not generally recommended for the treatment of IAV infections in immunocompetent patients if symptoms have been present for >48 h.

All acute viral infections are characterized by a rapid increase in the number of virus-infected cells upon virus encounter, followed by a gradual elimination of the infected cells by the innate and adaptive immune system. The onset of clinical symptoms is directly linked to the activation of the innate immune system, in particular the production of types I, II, and III interferons (IFN) [13,14]. Their concerted effects efficiently prevent virus infection of new cells within the respiratory tract (in the cases of IAV and SARS-CoV-2). This effect is rapidly augmented by humoral and cellular immune responses. Accordingly, the number of cells that continue to be newly infected decreases rapidly by many orders of magnitude within the following 5–7 days. Treatment with potent antiviral agents such as oseltamivir, which efficiently inhibits virus release and thus infection of new cells, can thus no longer influence the course of the disease. Patients with an impaired immune system are the only exception to this rule because of the prolonged course of their infections [12]. Importantly, oseltamivir is clinically effective when used at a time when the number of infected cells is still increasing, and it provides excellent protection against IAV when it is used before the onset of symptoms as part of prophylactic treatment regimens.

**Figure 1:**
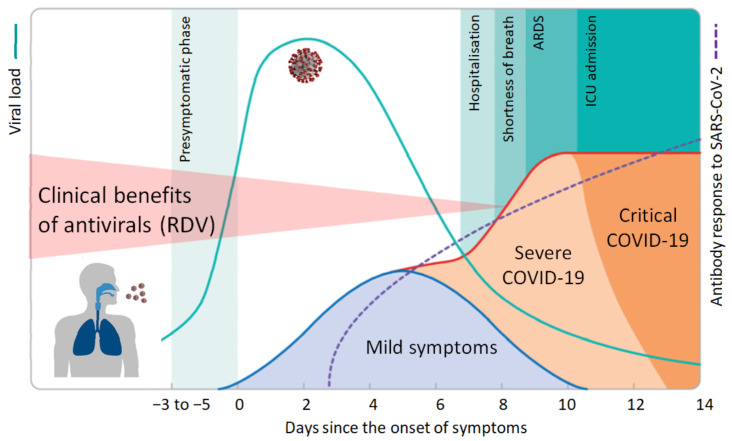
Course of COVID-19 and clinical benefits of antivirals. Following an incubation period of 3–6 days, SARS-CoV-2 infection generates a broad spectrum of clinical manifestations ranging from asymptomatic infection and mild illness to severe disease with high mortality. Viral load peaks around the day of symptom onset and rapidly declines thereafter. Accordingly, antiviral drugs like remdesivir (RDV) will only be effective early in infection when the number of new cells that become infected is still high. Antibody levels increase gradually and are commonly detectable after 7–14 days. Excessive immune responses, which may be curtailed by immunosuppressive agents like corticosteroids, lead to organ damage, intensive care admission, or death. Adopted from [19].

Similarly to IAV infection, the highest viral load and infectivity for SARS-CoV-2 are observed +/−1 day around the day of symptom onset [15]. Both the amount of infectious virus as well as the amount of viral RNA as measured by qRT-PCR decrease rapidly thereafter. Accordingly, the number of cells within the patient’s respiratory tract that are newly infected with SARS-CoV-2 declines sharply within a few days of disease onset. It is now well accepted that immunopathology plays a key role in severe COVID-19 [16]. Accordingly, treatment with corticosteroids, such as dexamethasone, improves survival in critically ill COVID-19 patients in the later stages of the disease [17]. It is important to note that corticosteroids are among the most potent pro-viral agents as they efficiently inhibit both the innate and adaptive immune response. The apparent benefits of steroids for COVID-19 patients provide further strong evidence that uncontrolled virus replication is no longer of major importance for disease outcome > 7–10 days after the onset of symptoms. A recent clinical trial on remdesivir reported that five days of treatment is not inferior to ten days of treatment [18]. While remdesivir may still have some clinical benefits during the first few days of treatment of COVID-19, inhibition of virus replication was no longer of clinical relevance a few days later, with patients requiring low-flow oxygen or corticosteroids (Figure 1).

An important difference between oseltamivir and remdesivir which has to be considered is their different modes of action: oseltamivir inhibits viral neuraminidase activity, thereby interfering with the release of new virus particles and infection of new cells. Viral gene expression and replication is not affected. In contrast, remdesivir reversibly inhibits the viral RNA-dependent RNA polymerase, which is required for both viral genome replication and expression of all viral mRNAs [3,9]. Remdesivir thus interferes with a much earlier step in productive infection. This may interfere with viral immune evasion and increase the visibility of the infected cells to the host immune system. However, when used in the late stages of infection, the release and infectivity of the already produced viral genomes, proteins, and virions remain inherently unaffected. This emphasizes the necessity of commencing antiviral treatment (very) early during SARS-CoV-2 infection.

Unfortunately, the key question of whether remdesivir has sufficient antiviral activity against SARS-CoV-2 for successful preemptive therapy or in early disease still remains unanswered. The observed shortening of the time to recovery observed in clinical studies provides strong evidence that it efficiently inhibits SARS-CoV-2 replication in COVID-19 patients. This is further supported by a preclinical study that demonstrated the efficacy of remdesivir in rhesus macaques when used 12 h after inoculation [20]. In addition, a recent case report of a COVID-19 patient without humoral immunity due to a prototypic genetic antibody deficiency X-linked agammaglobulinemia highlighted the excellent antiviral potency of remdesivir when used in a suitable clinical setting. In two independent 10-day courses of treatment, remdesivir reproducibly resulted in a rapid > 10^6^-fold decrease in viral load. After a rebound in viral load after the first 10-day course, this eventually led to clinical resolution and viral clearance in this persistently infected patient [21]. This single case report clearly demonstrates the high clinical efficacy of remdesivir against SARS-CoV-2. It thereby highlights that the clinical studies that have so far been conducted on remdesivir were poorly suited to the determination of its clinical effectiveness.

In general, the following therapeutic approaches for SARS-CoV-2 can be considered: (i) post-exposure prophylaxis (PEP) during the incubation period after documented SARS-CoV-2 exposure, (ii) early treatment after confirmed SARS-CoV-2 infection without any or with early symptoms, or (iii) treatment of advanced COVID-19. Antiviral approaches are most effective in the first two settings. Currently, no drug is approved for SARS-CoV-2 PEP. While hydroxychloroquine did not demonstrate any efficacy in a PEP setting [22], MK-4482 demonstrated efficacy for transmission prophylaxis in an animal model [23] and the first data in humans has been made available [24]. Clinical trials for other drugs such as AZD7442 (NCT04625972) are still ongoing. Other treatment options for these individuals have been explored with novel data demonstrating the efficacy of convalescent plasma in early infection in preventing severe COVID-19 in the elderly [25] when used early in the disease stage of COVID-19. Furthermore, monoclonal neutralizing antibodies (mAB) that act against the SARS-CoV-2 spike protein may contribute to the management of COVID-19 at early stages. Bamlanivimab (LY-COV555) was found to significantly reduce hospitalization rates [26], including when used for PEP [27], and was recently licensed by the United States Federal Drug Administration for use in COVID-19 outpatients in the early stages of the disease. However, due to the risk of rapid loss of efficacy, the emergency use authorization for mAB monotherapy of Balanivimab, but not the combination therapy with Etisivimab, was revoked [28]. Moreover, the monoclonal antibody combination of Casirivimab and Imdevimab demonstrated an accelerated decrease in the viral load upon treatment [29]. Currently no direct head-to-head studies of antiviral treatment and the use of mABs for early-stage SARS-CoV-2 or COVID-19 are available.

During the first year of the pandemic, the majority of COVID-19-related deaths resulted from large outbreaks in residential care homes. These were commonly associated with death rates of 10–20% and higher [30]. Due to their age and comorbidities, the elderly commonly succumb to COVID-19 within two weeks. However, when such outbreaks were detected, many infected individuals were still asymptomatic or within the first 1–2 days of symptoms. The rapid provision of intravenous remdesivir for 3–5 days by medical intervention teams to all PCR-positive residents could have drastically reduced mortality rates. Rapid initiation of antiviral treatment with remdesivir in high-risk individuals, defined as those aged > 70 years, immediately upon qRT-PCR-based confirmation of infection could potentially have saved many lives. Importantly, remdesivir has shown an excellent side effect profile and can thus be employed without major risk, even in the elderly. However, remdesivir has not yet been tested or licensed to treat asymptomatic or early-stage SARS-CoV-2 infections in individuals who are at >10% risk of COVID-19-related death. 

The availability of vaccines heralds the end of the pandemic. However, it will take many months, if not years, until herd immunity is achieved worldwide, and spike protein mutations of virus escape variants make herd immunity by effective vaccination even more challenging. Furthermore, not everyone will be vaccinated, and the role of vaccine non-responders is still unclear. With lockdowns and contact restrictions presumably coming to an end in early summer 2021 in the western hemisphere, we will inevitably see an increasing number of infections in children and non-vaccinated or unsuccessfully vaccinated adults next winter. With vaccination progressing rapidly particularly in the residential care homes, the number of high-risk patients that can be identified very early in infection or even prior to disease onset is rapidly decreasing. Similar to IAV infection, more and more patients will thus only be diagnosed > 48 h after the onset of symptoms. In September 2020, Gilead initiated a phase III clinical trial (NCT04501952) to evaluate the efficacy and safety of remdesivir treatment of early stage COVID-19 in an outpatient setting. However, in April 2021, Gilead made the decision to stop recruitment for this study based on changes in the COVID-19 landscape, the necessity of multiple day intravenous infusion treatment, and promising oral antivirals against SARS-CoV-2 in clinical trials. Patients already enrolled in the study will continue to be followed and the study remains blinded. We may thus soon learn whether remdesivir could have been a major game changer by substantially reducing COVID-19-related deaths in residential care homes.

## 3. Conclusions

Any antiviral agent will be most effective when applied early in SARS-CoV2 infection as it can then interfere with multiple rounds of virus replication. A key take-home message to ensure effective control of future pandemics is that any novel antiviral agent entering human trials should first be tested in a post-exposure setting, id est in PCR-positive individuals prior to the onset of symptoms. Here, clinical efficacy can be demonstrated (i) much more rapidly, (ii) based on a reduction in infection-related death (rather than a shortening of disease duration by a few days) and (iii) based on a much smaller number of patients, for example, in 150–200 inhabitants of a single residential care home that is experiencing a major outbreak associate with a 10–20% death rate. Only then should new antivirals be tested for clinical efficacy in more advanced viral disease stages.

## Data Availability

Not applicable.
